# CASME II: An Improved Spontaneous Micro-Expression Database and the Baseline Evaluation

**DOI:** 10.1371/journal.pone.0086041

**Published:** 2014-01-27

**Authors:** Wen-Jing Yan, Xiaobai Li, Su-Jing Wang, Guoying Zhao, Yong-Jin Liu, Yu-Hsin Chen, Xiaolan Fu

**Affiliations:** 1 State Key Laboratory of Brain and Cognitive Science, Institute of Psychology, Chinese Academy of Sciences, Beijing, China; 2 University of Chinese Academy of Sciences, Beijing, China; 3 Center for Machine Vision Research, Department of Computer Science and Engineering, University of Oulu, Oulu, Finland; 4 TNList, Department of Computer Science and Technology, Tsinghua University, Beijing, China; University of Lincoln, United Kingdom

## Abstract

A robust automatic micro-expression recognition system would have broad applications in national safety, police interrogation, and clinical diagnosis. Developing such a system requires high quality databases with sufficient training samples which are currently not available. We reviewed the previously developed micro-expression databases and built an improved one (CASME II), with higher temporal resolution (200 fps) and spatial resolution (about 280×340 pixels on facial area). We elicited participants' facial expressions in a well-controlled laboratory environment and proper illumination (such as removing light flickering). Among nearly 3000 facial movements, 247 micro-expressions were selected for the database with action units (AUs) and emotions labeled. For baseline evaluation, LBP-TOP and SVM were employed respectively for feature extraction and classifier with the leave-one-subject-out cross-validation method. The best performance is 63.41% for 5-class classification.

## Introduction

A micro-expression is a brief facial movement which reveals a genuine emotion that a person tries to conceal [1]–[3]. In addition, micro-expressions might be unaware and/or uncontrollable to the actor, thus may provide effective clues for detecting lies. Therefore, micro-expression recognition has many potential applications such as clinical diagnosis and interrogation. In the clinical field, micro-expressions may be used for understanding genuine emotions of the patients and promoting better therapies. For instance, Ekman [4] analyzed an interview video of a patient stricken with depression and that patient displayed a desperate micro-expression which could predict a commit suicide. During an interrogation or interview, micro-expression discloses what the interviewee really feels and thus helps further investigation. For instance, a micro-expression of scorn from Kato Kaolin's testimony in the O.J. Simpson trial betrayed his genuine feelings [5].

A micro-expression is featured by its short duration. The generally accepted upper limit of the duration is 1/2 s [3], [6]. These fleeting facial expressions, as observed, are usually low in intensity – it might be so brief for the facial muscles to become fully-stretched with suppression. Because of the short duration and low intensity, it is usually imperceptible or neglected by the naked eyes [1]. To better analyze micro-expressions and to help reveal people's feelings, an automatic micro-expression recognition system is in great need.

Automatic facial expression recognition is booming. Researchers had developed many algorithms and the accuracy has reached over 90% for the posed six basic facial expressions (anger, disgust, fear, happiness, sadness and surprise). On the contrary, studies on automatic micro-expression recognition have just started recently and only several pieces of work were available. Shreve et al. [7] divided the face into sub-regions (mouth, cheeks, forehead, and eyes) and calculated the facial strain of each sub-region. The strain pattern in each sub-region is then analyzed to detect micro-expression from the video clips. Pfister et al. [8] proposed a framework using temporal interpolation model and multiple kernel learning to recognize micro-expressions. Polikovsky et al. [9] used a 3D-gradient descriptor for micro-expression recognition. Wu et al. [10] developed an automatic micro-expression recognition system by employing Gabor features and using GentleSVM as the classifier. Wang et al. [11] treated a gray-scale micro-expression video clip as a 3rd-order tensor and utilized Discriminant Tensor Subspace Analysis (DTSA) and Extreme Learning Machine (ELM) to recognize micro-expressions. Ruiz-Hernandez and Pietikäinen [12] proposed to encode the Local Binary Patterns (LBP) using a re-parametrization of the second local order Gaussian jet to generate more robust and reliable histograms for micro-expression representation.

The success in conventional facial expression recognition largely rely on sufficient facial expression databases, such as the popularly used CK+[13], MUG [14], MMI [15], JAFFE [16], Multi-PIE [17], and also several 3-D facial expression databases [18], [19]. In contrast, there are very few well-developed micro-expression databases, which hindered the development of micro-expression recognition research. Eliciting micro-expression is difficult because it only presents at certain situations when an individual tries to suppress felt emotions but fails. According to Ekman, when a person tries to conceal his or her feelings, the true emotions would leak quickly and may be manifested as micro-expressions (Ekman & Friesen, 1969). In the early years, researchers constructed high-stakes situations to elicit micro-expressions, such as by asking people to lie about what they saw in video episodes [20], and by constructing crime scenarios (mock theft) and opinion scenarios (lie about own opinions) [21]. Micro-expressions elicited in these studies were commonly confounded by other facial movements (such as conversational facial movements), which are irrelevant to emotions and may “contaminate” the performance of the currently-not-robust micro-expression analysis algorithms. Some researchers developed small databases of micro-expressions by asking participants to pose facial expressions quickly [7], [9]. However, these posed “micro-expressions” are different from the spontaneous ones [2], [3]. Therefore, spontaneous micro-expression databases are necessary for academic research and practical applications. It was found that watching emotional video episodes while neutralizing faces is an effective method to elicit spontaneous micro-expressions without many irrelevant facial movements [3], [22]. Until now, there are only four micro-expression databases and only two of them contain spontaneous micro-expressions, as briefly summarized in Table 1.

**Table 1:** The current micro-expression databases.

	USF-HD [7]	Polikovsky's database [9]	SMIC database [22]	CASME database [23]
**subjects**	/	10	16 valid*	19 valid
**samples**	100	42	164	195
**Frames per sec.**	30	200	100	60
**Posed/spontaneous**	Posed	Posed	spontaneous	spontaneous
**AU labels**	No	No	no	yes
**Emotion class**	6	6	3	7
**remarks**	Their criteria set for micro- expressions (2/3 s) is longer than most accepted durations	/	Emotions are classified as positive, negative and surprise	Tense and repression in addition to the basic emotions

* Since not all of the participants showed micro-expressions, the recruited were more than the valid.

The first reason for developing a new database is that current spontaneous micro-expression databases are too small to provide sufficient samples. A large number of samples is needed for robust automatic micro-expression recognition research and related applications. We aim to provide more samples for training and test.

The second reason is that the video quality of current micro-expression databases cannot satisfy the need for micro-expression analysis. Micro-expression is brief in duration and low in intensity, which makes it difficult to detect and recognize. Higher spatial and temporal resolution may help to train the algorithms (which remains unclear). Compared with the 100 fps in SMIC and 60 fps in CASME, the sampling rate of CASME II is 200 fps, aiming to provide more detailed information on the facial muscle movements. In addition, compared with the face size of about 190 pixels×230 pixels in SMIC and about 150 pixels×190 pixels in CASME, the samples in CASME II have a larger face size about 280 pixels×340 pixels.

Third, we tuned the instruction in elicitation to increase the variety of the micro-expressions. Ekman (1969) mentioned two types of micro displays which may leak individual's genuine feelings: “The time-reduced full affect micro displays (i.e. micro-expressions) may well be those which the ego is not aware of, while the squelched micro displays may be those which the ego senses and interrupts in mid performance.” The elicitation paradigm used by Li et al. [22] and Yan et al. [23] only focused on the former, i.e. asking participants to fully suppress facial movements. The other type of micro-expression elicitation paradigm, i.e. asking participants to suppress facial movements when they are self-aware, is not used for collecting micro-expressions. It is possible that these two types of micro-expressions have different dynamic features. We aim to give participants different instructions to elicit both types of micro-expressions in order to increase the variety of our data.

This paper consists of two parts. In the first part, we introduce the procedure of developing CASME II, including methods of eliciting micro-expressions and how we select samples for the database. In the second part, we evaluate this database with Local Binary Patterns from Three Orthogonal Planes (LBP-TOP) for feature extraction and Support Vector Machine (SVM) for classification.

## Methods: Elicitation and analysis

### The method of micro-expression elicitation

#### Ethics statement

The experimental procedure was approved by the IRB of the Institute of Psychology, Chinese Academy of Sciences. The participants signed informed consent and had the right to quit the experiment at any time. The subject of the photograph has given written informed consent, as outlined in the PLOS consent form, to publication of their photograph.

#### Participants

Thirty-five participants were recruited, with a mean age of 22.03 years (Standard Deviation (SD) = 1.60) in the study. They all signed informed consent and had the right to quit the experiment at any time.

#### Apparatus

Four strictly selected LED lamps (to avoid possible flickering light caused by alternating current, 50 Hz) under umbrella reflectors (to focus the light on the participant's face) are placed to provide steady and high-intensity illumination. We designed such a setup because flickering light usually appear in high speed recordings (such as those in SMIC) and the video recorded usually turn out to be dark and noisy. We used Point Grey GRAS-03K2C camera to capture participants' faces, with the resolution at 640×480 pixels. “Raw 8” mode is used to reach 200 fps. We saved recordings as MJPEG format without any inter-frame compression.

#### Materials

Videos episodes with high emotional valence proved to be effective materials for eliciting micro-expressions [3], [22], [23]. Video episodes have a relatively high degree of ecological validity (Gross & Robert, 1995) and are usually better than pictures in term of emotional valence. Furthermore, video episodes are lasting and dynamic emotional stimuli, making inhibition more difficult (Yan et al., 2013). We used video clips similar to those for eliciting micro-expressions in CASME, but several invalid episodes were removed and several new ones were added. 20 participants rated the video episodes by choosing one or two emotion key words from a list and rating the intensity on a 7-point Likert scale (0 as the lowest and 6 as the highest). If a certain basic expression (e.g. happiness) was chosen by one third of the participants or more for a video episode, that emotion would be assumed as the principal emotion(s) of the video episode. There are individual differences of the subjective feelings at the same video episodes. Meanwhile, one episode may elicit several emotions, which is clearly exemplified by episode #12 and #17. Details of selected video episodes and their corresponding rating scores are listed in Table 2.

**Table 2:** Video episodes used for eliciting micro-expressions and participants' rating scores.

Episode NO.	Content	Duration	Main elicited emotion(s)	Rate of selection	Mean score (intensity)
1	Ad of 7 Up	2′52″	happiness	0.69	3.27
2	Sports fault	1′18″	happiness	0.71	3.60
3	Jokes on names	51′	happiness	0.70	3.14
4	Larva (animation)	1′32″	happiness	0.64	4.43
5	Larva 2 (animation)	1′32″	happiness	0.94	3.93
6	Jokes on stool	1′28″	disgust	0.81	4.15
7	Tooth extraction	1′7″	disgust	0.69	4.18
8	Eating worm	1′35″	disgust	0.78	4.00
9	A brazen man	1′34″	disgust	0.81	3.23
10	Operation for Near-sighted eye	1′56″	fear	0.63	2.90
11	Ring(Horror film)	2′4″	fear	0.67	2.83
12	Meat grinder	2′25″	disgust (fear)	0.60(0.33)	3.78(3.6)
13	Final destination(movie)	1′43″	fear	0.5	3.72
14	Roots and Branches (Movie)	2′26″	sadness	1.00	4.27
15	Derek Redmond in 1992 Olympics game	1′31″	sadness	0.71	4.08
16	A girl killed by cars	1′57″	sadness	1.00	5.00
17	Train accident and officer's dereliction	1′57″	anger (sadness)	0.69 (0.61)	4.33(4.88)
18	Torturing dog	1′37″	anger	0.75	4.67
19	Beating a pregnant women	2′25″	anger	0.94	4.93

#### Procedure

**Figure 1:**
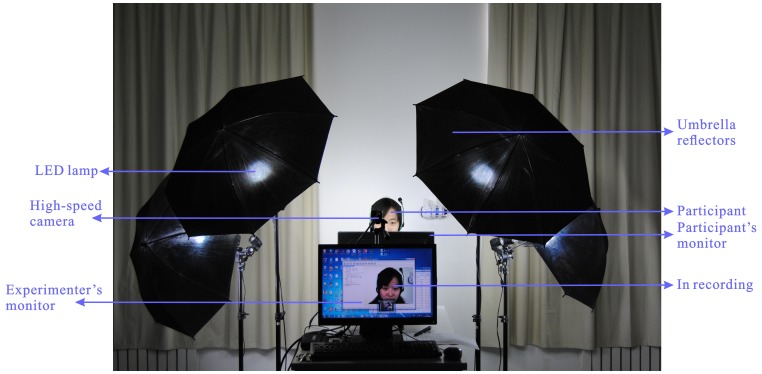
Acquisition setup for elicitation and recording of micro-expressions.

To elicit such facial expressions, we induced participants to experience a high arousal and promoted a motivation to disguise. Similar procedures were conducted as for building SMIC and CASME (see Figure 1). The difference is on the instructions given to the participants: 18 participants were asked to keep neutralized faces when watching video clips while 17 participants only tried to suppress the facial movements when they realized there was a facial expression. This design was for eliciting two types of micro-expression [1] as explained above in the Introduction. The participants were asked to watch the video clips in front of a screen and avoid any body movement. The experimenter monitored participants' face on-line from the other monitor. This setup helped the experimenter to pre-define some habitual movements which will be verified by the participant after each video play finished.

### Sample selection and category labeling

Two coders were involved in the analysis of the micro-expressions. We processed the raw video recordings in following steps to select micro-expressions:

#### Step 1

Remove the irrelevant facial movements. There is a large quantity of facial movements in the recordings but many are obviously irrelevant, such as head movements, pressing the lips when swallowing saliva or other habitual movements (such as blowing the nose). All the unemotional movements were ruled out.

#### Step 2

Pre-select micro-expression candidates by restricting the to-be-analyzed samples. The coders browsed and spotted the onset and offset frames of each emotional movement clip and only those whose duration last less than *one second* were saved as micro-expression candidates for a further coding. Expressions that are too subtle to be precisely coded are also excluded.

#### Step 3

Candidate micro-expression clips were converted into frame sequences to facilitate precise labeling of onset and offset frames. By employing a frame-by-frame approach, the precise frame numbers of onset and offset of each sequence were decided, and only those met the selection criteria (total duration less than 500 ms or onset duration less than 250 ms) were chosen as the final micro-expression samples.

After the micro-expressions were selected, AU(s) were marked and emotion labels were given for each micro-expression by two coders. The reliability is 0.846, which is calculated by:
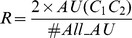
where #*AU(C_1_C_2_)* is the number of AUs on which Coder 1 and Coder 2 agreed and #*All_AU* is the total number of AUs in a micro-expression scored by the two coders. Afterwards, the two coders discussed and arbitrated the disagreements.

FACS [10] is an objective method for labeling facial movements in terms of component actions. Different groups may have different micro-expression classes but could have the same AU coding system. Unlike posed facial expressions, for which people were asked to generate several preset facial movements, the spontaneous micro-expressions in this database were elicited by strong emotional stimuli. Therefore, it is inappropriate to forcefully classify these micro-expressions into the same six categories as for ordinary facial expressions. For example, AU4 (frown) may indicate disgust, anger, attention or tense [3]. In CASME II database, the micro-expressions are labeled based on the AUs, participants' self-report, and the contents of the video episodes. We provided five main categories (as listed in Table 3).

**Table 3:** Criteria for labeling the emotions and the frequency in the database*.

Emotion	Criteria	N
**Happiness**	either AU6 or AU12	33
**Disgust**	one of AU9, AU10 or AU4+AU7	60
**Surprise**	AU1+2, AU25 or AU2	25
**Repression**	AU15 or AU17 alone or in combination	27
**Others**	Other emotion-related facial movements	102

* The emotion labeling are just partly based on the AUs because micro-expressions are usually partial and in low intensity.

Therefore, we also take account of participants' self-report and the content of the video episodes.

### Profile of the database

**Figure 2:**
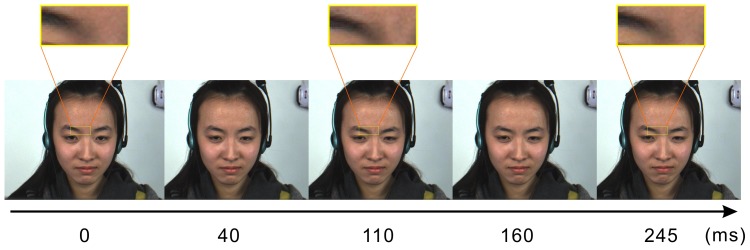
A demonstration of the frame sequence in a micro-expression. The apex frame presents at about 110-expression is 4+9 (with AU 17 kept almost unchanged), which indicates disgust. The three rectangles above the images show the right inner brow (AU 4) in “zoom in” mode. The movement is more obvious in video play than picture sequence.

The developed database called CASME II contains 247 micro-expression samples from 26 participants. They are selected from nearly 3,000 elicited facial movements. These samples are coded with the onset and offset frames, with action units (AUs) marked and emotions labeled and five main categories are provided, as listed in Table 3. The criteria employed to label emotion categories are also shown in Table 3, and one example of micro-expression clip is illustrated in Figure 2. Very subtle micro-expressions were removed because they are almost unable to code the onset and the offset (difficult to detect the turning points due to the almost imperceptible changes). The CASME II database has the following characteristics:

- The samples are spontaneous and dynamic micro-expressions. Baseline (usually neutral) frames are kept before and after each micro-expression, making it possible to evaluate different detection algorithms.
- The recordings have high temporal resolution (200 fps) and relatively higher face resolution (280×340 pixels).
- Micro-expression labeling is based on FACS investigator's guide [24] and Yan et al.'s findings [3]. The labeling criteria are different from the traditional 6 categories on ordinary facial expressions.
- The recordings have proper illumination without flickering light and with reduced highlight regions on the face.
- Some types of facial expressions are difficult to elicit in laboratory situations, thus the samples in different categories distributed unequally, e.g., there are 60 disgust samples but only 7 sadness samples. In CASME II, we provide 5 classes of micro-expressions.

## Database evaluation

### Preprocessing

Before running any formal experiment on this dataset, three steps of preprocessing were carried out on the raw sample clips that have been labeled. Let 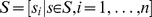 be the set of micro-expression clips. The *i*th sample 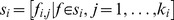, 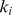 is the frame number of sequence 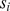.

First, a frontal face image M with neutral expression was selected as the model face. 68 facial landmark points of the model face 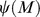 were detected using the Active Shape Model [25]. Second, the first frame of each micro-expression clip 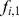 was normalized to the model face using a Local Weighted Mean (LWM) [26] transformation, and the transform matrix T is written as:
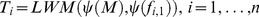
(1)Where 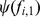 is the coordinates of 68 landmark points of the first frame of the micro-expression sample 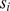. Then all frames of 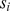 were normalized using the same matrix 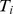. There are two reasons why we detected ASM landmark points only on the first frame but not on all frames. The first reason is that, since the time duration of a micro-expression is very short the rigid head movement within the duration can be neglected. The second reason is that the landmark points detected by ASM may be not accurate enough; if applied on a sequence of frames, there could be big deviation of locations of the same point even when the face did not move at all. The normalized image 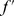 was computed as a 2D transformation of the original image:
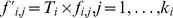
(2)

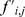
 is the *j*th frame of the normalized micro-expression sequence 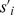.

Third, the eye coordinates 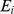 of the first frame of each normalized micro-expression sequence 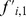 were located and then the face of each frame of 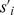 was cropped out using a rectangle determined by eye positions 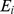.

### Experiments

**Figure 3:**
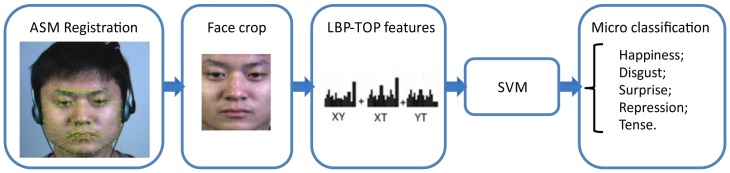
Block diagram of the micro-expression classification.

We carried out a micro-expression recognition experiment on the samples preprocessed by the steps described in the previous section. LBP-TOP features were extracted to describe micro-expressions from a spatiotemporal point of view, and SVM was employed as the classifier to provide baseline performance for the future evaluation of our database (Figure 3). The details are described below.

### Methods

#### Local Binary Patterns from Three Orthogonal Planes (LBP-TOP)

**Figure 4:**
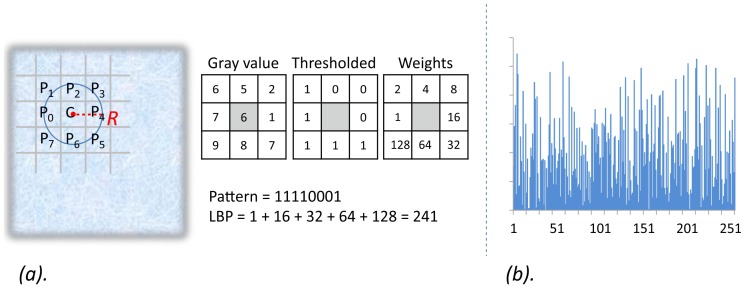
The LBP operator and its histogram.

The conventional LBP method has been demonstrated to be effective for describing 2D textures of static images [27]. Considering one pixel C in the image, LBP operator describes its local texture pattern by comparing and thresholding the gray values of its neighboring pixels. As illustrated in Figure 4(a), for the center pixel C with its P neighboring pixels with the radius R, the LBP value is calculated as:
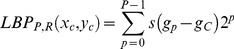
(3)Where 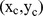 are the coordinates of the center pixel C and 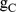 is its gray value, and 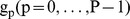 is the gray value of its pth neighboring pixel on the radius R. 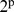 is the weight corresponding to the neighboring pixels locations, which is used to transform a binary pattern string to a decimal index for this pattern. The function 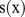 is a sign function defined as:
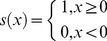
(4)


Given an input image of size M by N, the LBP value *b* of each pixel could be calculated using the above two equations. Then we can form a feature vector to represent the input image by calculating the histogram distribution of all of its LBPs:
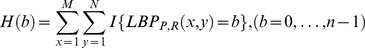
(5)Where n denotes the total number of interested patterns, and:
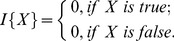
(6)


One example of LBP histogram is illustrated in Figure 4(b). More details about LBP please refer to [27].

As an extension of the basic LBP idea, Local Binary Pattern on Three Orthogonal Planes (LBP-TOP) was proposed by Zhao *et al* [28] for dynamic texture analysis in the spatial-temporal domain.

**Figure 5:**
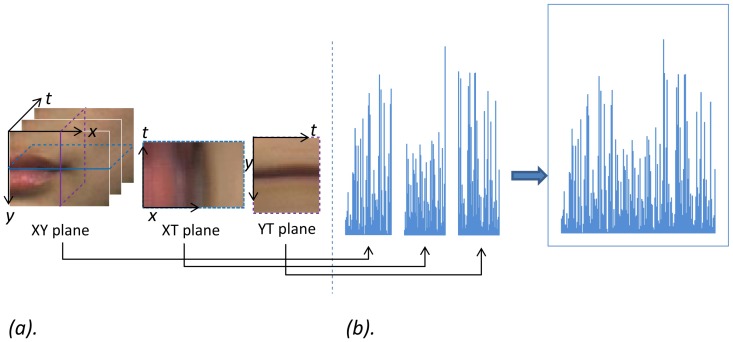
The texture of three planes and the corresponding histograms. (a) XY, XT and YT planes of a micro-expression sample, (b) concatenated LBP-TOP feature.

Given a video sequence of time length T, usually it can be thought as a stack of XY planes along the time axis T, but it can also be thought as a stack of XT planes on axis Y, or a stack of YT planes on axis X. The XT and YT planes provide information about the space-time transitions. Taking micro-expression as an example, the XT and YT planes contain information of how the gray values of a row or a column of pixels change along the time dimension; if the input data volume is of a mouth corner, they describe the mouth movement as dynamic texture in the XT or YT plane accordingly (see Figure 5(a)).

The basic idea of LBP-TOP is the similar as LBP, which is to use an index code to represent the local pattern of each pixel by comparing (and thresholding) its gray value to its neighbors' gray values. To include information from 3D spatial-temporal domain, LBP code is extracted from the XY, XT and YT planes of each pixel, respectively. Then one histogram of each plane is formed by using equation (3) and (4) and then concatenated into a single histogram as the final LBP-TOP feature vector. Note that we can set different values for the radius of X, Y and T axis as 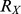, 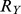 and 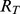, so finally we can have elliptical sampling than circular sampling. The procedure of extracting LBP-TOP feature histogram is demonstrated in Figure 5(b).

### Results

The radii in axes X and Y vary from 1 to 4. The radius of T varies from 2 to 4 (we don't consider T = 1 because the samples are in 200 fps where the change of two neighboring frames is little). The number P of the neighboring points in the XY, XT and YT planes was set as 4. SVM was used as the classifier. For this baseline evaluation, we selected 5 classes of the micro-expressions–happiness, surprise, disgust, repression and others–for training and test. Considering samples unequally distributed in the five selected classes, leave-one-subject-out cross validation was applied. The LBP-TOP features were extracted in 5×5 blocks. The performance is shown in Table 4. The best performance is 63.41% where the radii are 1, 1 and 4 for XY, YT and XT planes respectively.

**Table 4:** Performance on recognizing 5-class micro-expressions with LBP-TOP features extraction and leave-one-out cross-validation.

R_X_	R_Y_	R_T_	5×5 blocks (%)
1	1	2	63.01
1	1	3	62.60
**1**	**1**	**4**	**63.41**
2	2	2	61.38
2	2	3	61.79
2	2	4	62.20
3	3	2	58.54
3	3	3	61.79
3	3	4	58.55
4	4	2	58.94
4	4	3	61.38
4	4	4	60.57

## Discussions and Limitations

There are still some limitations regarding the elicited micro-expressions and this database:

First, the labeling for these spontaneous micro-expressions is not very satisfactory. The materials for eliciting micro-expressions are video episodes which are complex stimuli and can have different meanings to different people. For example, the scene of chewing worms is not always disgusting—some participants actually reported “funny” or “interesting”. Categorizing the micro-expressions simply based on AUs (especially for AU combinations that haven't been defined) requires further in-depth studies. Participants are required to control and suppress their facial expressions, which often makes spontaneous micro-expressions appear partial and sometimes with only a single AU (also see Porter, 2008; Yan et al., 2013). Even though we considered the AU combinations, the content of the video episodes and the participants' report, the emotion labels for these micro-expressions remain debatable.

Second, the micro-expressions in this database are elicited under one specific lab situation and may not cover the micro-expressions elicited under other situations. Micro-expressions that occur when people are under an police interrogation or lying to their boss, may be different from those we observe in a laboratory situation. Up to now, very few studies have investigated the micro-expressions elicited in different situations. Future studies should be conducted to explore the variations of micro-expressions in different situations.

Third, it's unclear which method is most suitable for classifying micro-expressions. The micro-expressions have the following characteristics which are different from the ordinary facial expressions in the following ways: (1) low-intensity facial movements and (2) partial facial expressions (fragments). Therefore, previous methods that were suitable for classifying ordinary facial expressions may not work well for micro-expressions. We provided this database and a baseline evaluation, so future research may compare with our initial results.

Fourth, the participants came from a limited range of age. Those who participated in the elicitation section are young people. Most of them are university students. It would be better if the database contain samples from models at various ages.

## Conclusions

This paper reviews the previously developed micro-expression databases and presents a new and improved one. This new spontaneous micro-expression database, CASME II, includes 247 micro-expression samples which is larger than previous micro-expression databases. The micro-expressions were elicited in a well-controlled laboratory environment and they were recorded by a high-speed camera (200 fps) so the temporal resolution is much higher than previous spontaneous micro-expression databases. For the spatial resolution, the size of the faces is larger than previous spontaneous micro-expression samples; as larger scale of images helps to reveal tiny changes, which would benefit feature extraction and futher better classification. The recordings have proper illumination without flicking light and with reduced highlight regions on the face. These samples were coded with onset and offset frames as well as AUs and emotion labels. We also reported the baseline results of evaluation using a combination of LBP-TOP and SVM.

Micro-expression analysis is a new research area. Starting from a controlled laboratory environment can make the database well focused on the subtle expressions and rule out irrelevant factors. We provide the CASME II database as a benchmark for the future exploration of potential micro-expression analysis algorithms that are suitable for detecting subtle facial muscle movements for emotional states recognition. In the future, we will extend our research to a more natural environment for expression and micro-expression analysis in natural conversation and interaction. The CASME II database is online public available now for testing (see http://fu.psych.ac.cn/CASME/casme2-en.php for details).
